# Elements of Medicine, Vol. I. On Morbid Poisons

**Published:** 1837-07-01

**Authors:** 


### Elements of Medicine. Vol. I. On Morbid Poisons. By Robert Williams, M.D. Senior Physician to St. Thomas's Hospital.

This is a work of great merit, and deserves public encouragement. The volume embraces Typhus, Scarlatina, Morbilli, Variolæ, Varicella, Erysipelas, Pertussis—each of which deserves a short article in our Journal.

In a well-written Introduction of 22 pages, Dr. Williams reviews the subject of morbid poisons generally, and has collected from the best sources, ancient and modern, foreign and domestic, whatever is known (that is worthy of record), respecting the laws by which they are governed, in their operations on the animal economy. In this part the author has introduced a good deal of original matter, in the way of comment or practical observation. We present the note at page 7, as a specimen.

"It is a popular prejudice, that if we would prevent the action of mercury on the system, we must give a purgative draught the next morning, which will carry it off. Dr. Fordyce brought this opinion to the test of experiment. He selected in the wards of St. Thomas's Hospital a given number of patients, whom it was thought necessary to bring under the influence of mercury. To one half of these persons he gave five grains of calomel every night, and followed it up the next morning by a purgative draught. To the other half he gave the same quantity of calomel, but omitted the purgative draught. The former patients were much sooner under the influence of mercury than the latter; a result which can only be accounted for on the supposition that the debility occasioned by the purgative medicine must have rendered the constitution more susceptible of the mercurial sialagogue." 7.

In respect to the modus agendi of morbid and other poisons in general, he contends for the doctrine of absorption of the foreign matters into the torrent of the circulation, and their direction to particular structures afterwards, where they operate according to their specific affinities.

"The poison having once been introduced into the circulating torrent, in what manner does it act? Dr. Addison and Mr. Morgan are of opinion, that, on entering the bloodvessels, it makes an impression on the nerves supplying the inner coat of the vessel, and is then instantly destroyed; while the impression made on the nerve is conveyed to the brain, whence it is reflected to the parts secondarily affected. The more recently received opinion, however, is, that poisons are conveyed unchanged, or only modified, to the parts on which they more specifically act; and certainly the weight of evidence is greatly in favour of this latter hypothesis. For it is quite impossible to explain the fact of iodine and a variety of other substances, being found in the urine, and other secretions of the body, or else thrown off by the lungs, without inferring that these poisons have been carried to those parts either in substance, or else resolved into their elements—a state which, for anything we know, may be equally deleterious with the original compound. Some substances, also, have been found unchanged and embedded in the bones as madder. There is, likewise, no fact better proved in physiology, than that the different tissues of the body are acted upon by particular stimuli only. Thus the retina is insensible to sound—the tympanum to light. The secretions of the bladder and intestines produce healthy actions of those viscera; but if through disease these parts communicate, and their contents mingle, gangrene, and the saddest results are the consequence. There is little question, consequently, but that poisons are conveyed to the parts on which they act, and produce their effects by immediate contact, and not by the intervention of the brain; for the brain has been removed in animals poisoned by strychnine, and yet the phenomena of tetanus have resulted." 20.

Our author, however, is far from denying that the nerves play an important part in the agency of poisons. Prussic acid, and even arsenic (when in large doses), will destroy life before there can be absorption at all—and consequently it must be by the deleterious impressions which they make on the sentient extremities of the nerves. The following is Dr. Williams's distribution of diseases produced by morbid poisons.

"*The Diseases that are both Contagious and Infectious are*—

| | |
|---|---|
| Typhus. | Varicella. |
| Scarlatina. | Erysipelas. |
| Morbilli. | Pertussis. |
| Variolæ. | |

*The Diseases that are Simply Contagious are*—

| | |
|---|---|
| Variolæ Vaccinæ. | Pestis. |
| Syphilis. | Cellulitis Venenata. |
| Gonorrhœa. | Cellulitis Farciminosa. |
| Hydrophobia. | Tinea: |

*The Diseases that are neither Contagious nor Infectious, but depend on the agency of certain Miasmata, are*—

| | |
|---|---|
| Febris Palustris. | Dysenteria Palustris. |
| Cholera Indica. | |

This catalogue might perhaps admit of some additions, as Catarrh, Influenza, Angina Parotidæa, &c.; but it has been thought preferable, until the arrangement shall have received the approbation of the profession, not to introduce any disease that might give rise to controversy." 23.

We were gratified to find that an enlightened hospital physician, whose public practice is in the Borough, has discarded the late epidemic cholera from among the contagious, or even infectious diseases. The most decided contagionists and alarmists of 1832, who intimated little less than that the anticontagionists deserved burning at the stake, are now silent and chap-fallen, seeing that their darling prophecies of Indian cholera becoming domiciliated in England, are all vanished into air! We will not be so uncharitable as to suppose that the Cholera-board-men—in other words, the red-hot contagionists would rejoice in seeing their prophecies verified. We believe they would feel quite otherwise than rejoiced:—for they were woefully afflicted with *Cholera-phobia*, and some of the heads of departments, to our knowledge, kept at a very respectful distance from cholera patients, whenever they could, with decency, do so? But we need not now dwell on days of Lang Syne.

### TYPHOID POISON.

Typhus is defined by our author a "continued febrile disorder, having no intermissions:—it runs a course of very varied length, and is both infectious and contagious." He goes on to say, that "there is but one simple continued fever in this country—and that is caused by the agency of typhoid poison." The ancients, living in warm climates, were unacquainted with this fever. Willis, he thinks, was the first physician to distinguish between typhus and the malarious or paludal fevers. Morton makes the same distinction. Our author then traces the history of typhus down from the Cambridge assizes, 1521, to the fatal event of the Old Bailey in 1780, and finally agrees with Pringle in believing that the fevers of different years are uniform in character, though differing in intensity, and—

"That all simple continued fevers in this country, by whatever name distinguished, as jail fever, hospital fever, low fever, nervous fever, brain, putrid, or bilious fever, are mere varieties of the same continued and contagious disease—typhus fever." 30.

The remote cause of this poison is, at present, hidden from our sight and senses. The two most feasible hypotheses, he thinks, are the emanations from the persons of healthy, or unhealthy, but crowded bodies of people—and, secondly, the effluvia from marshes, that is from decomposing vegetable matters. Another hypothesis, however, to which our author is inclined to give assent, is this, that the poison of typhus is always diffused through the atmosphere, but only comes into operation when the human frame is predisposed thereto, by other deleterious causes, as heat, cold, mechanical injuries, &c.

"It results, therefore, that we must admit the typhoid poison to be generally diffused through the atmosphere; for typhus fever, in those countries to which it is a native, is equally present in the crowded and populous city, and in the lone and solitary hut; and, in proportion to the population, is as frequent on the mountain as on the plain. The poison, also, may be affirmed to exist at all periods of the year, for no season is free from its ravages; but it varies greatly in quantity or intensity, the fever being mild or fatal, sporadic or epidemic in different years." 33.

As to the power of this fever communicating itself from one individual to another, our author has no doubt. But as we find an immense majority of the fevers of this country apparently non-contagious, many will be disposed to question the truth of their being all typhoid and contagious. That typhus itself is contagious, there can now be little doubt, and the proofs brought forward by Dr. W. are incontrovertible.

"The poison which has thus been proved to be the cause of typhus fever, is subjected to certain laws. It is capable of being diffused through the atmosphere, and, according to its intensity, of producing disease at certain distances—it also infects fomites—has a predilection for certain victims—lies latent a given period, gives rise to a certain series of phenomena, and the course of those phenomena is modified by modes of treatment." 37.

All these points are illustrated and discussed by Dr. Williams, but the facts and arguments are familiar to our readers, and need not be adverted to in this place. Typhus disproves the Hunterian dogma, that two constitutional diseases cannot co-exist. It is often seen in conjunction with scabies, syphilis, erysipelas, &c.

"*Pathology*.—The typhoid poison having been absorbed and mingled with the blood, and the period of latency passed, it primarily induces certain derangements of the *functions* of the great nervous centres, and consequently of the many organs and systems they supply; derangements which constitute the phenomena of fever; as alterations of temperature—changes in the force or frequency of the pulse—disorders of the alimentary canal, with other concomitant affections. After a certain time, however, not yet accurately determined, the typhoid poison, in addition to the febrile phenomena, induces certain local lesions or alterations of structure in a limited number of organs or tissues of the body. The alterations of structure are, first, inflammation of the mucous membrane of some portion of the intestinal canal, which membrane is the great and primary, if not the constant seat of the action of the poison; secondly, inflammation of the membranes of the brain, which, though not constant, are frequent; thirdly, certain cutaneous eruptions, likewise frequent, but not constant; and lastly, inflammation of the substance or bronchial membrane of the lungs, or both, which occurs in a small number of cases only." 41.

This pathology is probably as near the truth as any that has been offered hitherto. Broussais and his disciples will consider their claim to superiority over Clutterbuck and his adherents, as strengthened, if not established by this declaration. There is, however, a very pertinent saving clause introduced by Dr. Williams, which cannot prove very palatable either to the gastro-enterite or encephalite party.

"The law, that fever precedes the great specific actions of the typhoid poison, is deduced from the fact, that there are many instances in which the patient has fallen in typhus, and yet no alteration in the structure of any has been traced: it is consonant also with the laws of other morbid poisons. The specific actions of the poisons of small-pox or of scarlet fever are not set up till after some days' preliminary fever; and the phenomena of intermittent fever precede frequently by many weeks or many months the occurrence of any local affection." 41.

This just brings us back to the commonly received opinion, that fever is a general disease, producing local effects, in a great number of instances, where life is not destroyed by the onset of the disease. The interval between the accession of the fever, and the commencement of the topical inflammations, cannot be precisely determined. Dr. W. thinks it is not great. Chomel had an opportunity of examining a case where the patient died on the seventh day, yet the follicles of the mucous membrane of the intestinal canal were found to be enlarged.

Sir John Pringle appears among the first to connect fever with gastro-enteritis. He examined ten patients who died of typhus, and came to the conclusion, that when the disease proves fatal, it generally terminates in actual mortification of some part—especially the intestines—or in abscess of the brain. This was in 1750. Ten years afterwards, Rœderer and Wagher, in Gottengen, found the intestines inflamed in the fever that prevailed among the French troops occupying that city. They considered this fever, however, as one *sui generis*, and drew no useful conclusions from it. In 1803, M. Prost drew the attention of the profession to the pathology of fever, stating that he had examined the bodies of upwards of 150 people who had died of "*fiévre ataxique*," and always found inflammation of the gastro-intestinal mucous membrane, with or without excoriation. In 1813, Messrs. Retil and Serres brought forth a work "Sur la Fiévre Entero-Mesenterique," the pathological peculiarities of which were inflammatory alterations in the mucous follicles of the ileo-cæcal valve and neighbouring parts. Broussais is passed over without notice by Dr. Williams—a most singular omission—and which we cannot explain. Bouillaud, Andral, Alison, Chomel, are mentioned as adding their quota to the illustration of fever pathology.

"When the typhoid poison sets up its great specific action on the mucous membrane of the alimentary canal, that of the cæcum, or ileo-cæcal valve, is in a great majority of cases the exclusive seat of the disease; but in a smaller number of cases the inflammation extends its ravages both upwards and downwards, from a few lines to many inches, from those points as from a centre. In a few instances, however, the colon or small intestines, or the stomach, is the exclusive seat of the disease, or the poison may involve any combination of those parts. The inflammation thus excited may attack the free surface, or the adherent surface of the mucous membrane, or it may fall on its most common seat of action, the mucous follicles, and attack these parts either separately or conjointly.

When the poison falls on the free surface of the mucous membrane of the alimentary canal, it excites diffused inflammation of the part, which may terminate either by resolution or by ulceration. Diffuse inflammation of the mucous membrane of the alimentary canal, in whatever part it occurs, is characterised by a deep venous colour, almost approaching to blackness. The substance of the membrane is also generally thickened, and its adherent surface usually, though not constantly, softened so that we can readily detach large portions of it by means of the handle of the scalpel.

Diffuse inflammation, though it sometimes terminates in resolution, yet in fever more frequently ends in ulceration; and the ulcers vary greatly in number, form, and character. There may be one or several, their form may be either regular or irregular, and their edges may be either elevated or depressed, indurated or broken down. If the ulceration proceeds, the muscular and serous tunics are involved, and a livid congested spot, seen through the peritoneal coat, marks the precise seat of the ulcer; and if the disease proceeds still further, the intestine bursts, and the patient dies of peritonitis. In a very small number of cases the specific action of the poison falls exclusively on the cellular tissue connecting the coats of the intestines, and, perhaps, principally on that of the colon. This inflammation usually terminates in the formation of a number of small abscesses, over which the mucous membrane softens and bursts, and then their contents are discharged into the intestinal canal. The appearance of this form of inflammation is that of a pustular disease, and has been compared to the small-pox. These abscesses sometimes burrow in an opposite direction, and the patient dies, as in the former case, from peritonitis." 47.

The typoid poison does not act exclusively, as some have imagined, on the mucous follicles. In one year, or in one epidemic, it may do so; in another it may act on every constituent part of the mucous membrane. When it acts on the glands, those of the ileo-cæcal valve are most frequently the seat of the mischief.

"When inflamed in the first degree, or that of serous inflammation, the gland, scarcely visible in health, becomes greatly enlarged in consequence of effusion into its substance, while the transparency of its tissues causes it to appear translucent, with a black point in its centre, which is the excretory duct. In children and others, when the fæcal matters are void of bile, the gland is colourless like a drop of water; but when much bile is present, it usually represents the appearance of a drop of amber.

The second stage of follicular inflammation is marked by the gland having lost the transparency of the first stage, and being reduced in bulk compared with the first degree of inflammation, though still much larger than in health.

It is now hard, prominent, white and opaque. The first and second stages of follicular inflammation may terminate by resolution.

The third and last stage of follicular inflammation is ulceration. In this case, the gland bursts, and its contents escaping, leave a deep depression in the mucous membrane, so that supposing the ulceration to occur in an elliptical patch, the unequal surface of the mucous membrane gives that part the appearance of being granulated. The appearance of the ulcer, whether of the glandulæ segregatæ or aggregatæ, are very various; sometimes their edges are something elevated, or broken down, and their base covered with a putrid sanies ; at others the edges are perpendicular, as if made by a punch, while the base of the ulcer is clean, exposing the muscular or peritoneal coat, which in a few cases bursts, and the termination of the disease is then by peritonitis. When the action of the poison is at length worn out, the ulcer granulates and heals, but a depression remains, denoting its seat, and the imperfect reparation of the part." 48.

M. Louis found the stomach more or less diseased in one half of the cases; but in these and in all the others, there was follicular disease of the cæcum and intestines.

" When the stomach is the seat of disease in fever, the poison commonly induces either to diffuse or ulcerative inflammation of the mucous membrane. The colour of this inflammation is a deep venous purple, which changes in a few minutes, after exposure to the atmosphere, to a bright arterial colour. The discoloured portion is also usually altered in texture, the villosities being broken down, and the surface more pulpy than natural; the mucous membrane also is thickened, and its cohesion impaired, so that it is more readily detached, and in larger proportions than in a state of health. The inflammation is usually partial, occupying the convexities of the folds, or the great cul de sac, or the orifices of the stomach : occasionally, however, the stomach is one uniform dull red." 49.

This diffuse inflammation may pass into the ulcerative, the ulcers assuming various forms and characters; but generally being small, and presenting a sharp edge with perpendicular sides, as if made by a punch. They are most frequent about the pyloric orifice, and in the great cul de sac.

Next to the intestinal canal, the brain and its membranes are the parts most frequently affected by the typhoid poison.

" It has been calculated that in five cases out of six the functions of the brain are more or less deranged in fever, while in the sixth case, though the powers of the mind may be enfeebled, yet they are in no sensible degree perverted; and even in those cases in which the delirium and general excitement show the functions of the brain to be most disturbed, still it has frequently happened that no trace of inflammation, or other alteration of structure, has been found in the brain or its membranes after death." 50.

It is remarked by our author that the science of pathology is not sufficiently advanced to enable us to determine whether mere injection of the cerebral vessels constitutes inflammation of the brain. We seldom see more than vascular turgescence of these parts in fever. The membranes, however, are much more frequently affected than the brain itself, and appear to be the more specific seat of the poison's action. The dura mater usually escapes; but the arachnoid and pia mater are liable to the different forms of inflammation, as the diffuse, the serous, the adhesive, and the purulent—all of which forms are not unfrequently seen in the same subject.

The organs, next in order of attack, are the lungs. The frequency, however, is very variable, according to the seasons of the year. "In this country, judging from the hœmoptysis and purulent expectoration so frequently met with during life, the bronchial membrane would seem to be the most frequent seat of the disease." But all calculations have been only distant approximations to the truth. The pulmonary texture itself is not so frequently affected as the mucous membrane. The skin is subject to more than one affection in fever—to petechiæ, sudamina, and, towards the close of the disease, to ulceration. The petechial eruption most usually appears from the 7th to the 9th day—sometimes much later.

The symptoms of typhus form a most complex problem, according as the poison acts on the three great nervous centres. Its varying intensity has induced pathologists to divide it into typhus mitior and gravior, together with several subdivisions. One of the most remarkable symptoms is the extreme prostration of strength, both moral and physical. But we need not dwell on the symptomatology of typhus, which is peculiar even to our tyros.

*Treatment.*—This is divided into the curative, dietetic, and preventive. Is it possible to arrest typhus in its march? Cold affusion and emetics were thought capable of suspending fever. This remedy is now generally laid aside for sponging—and this, too, is only palliative. As to emetics, they often do harm, where the mucous membrane of the stomach and bowels is in an irritable condition. They never can stop the action of a morbid poison, and they are fast falling into disuse. Bark and mercury have specific effects, no doubt; but they cannot be expected to stop the progress of a fever dependent on an aerial or terrestrial poison. Mercury, however, is an important medicine in conducting fever to a favourable issue. In short, there is no known antidote to the poison of typhus. Our author glances at the various modes of treating this disease, as advocated by Sydenham, Huxham, Pringle, Lind, Fordyce, and modern writers of England and the Continent. These need not detain us. The following passage contains judicious precepts.

"The great laws of fever that should guide us in the treatment of this disease are, first, that it has a course to run; and secondly, according to the idiosyncrasy of the patient, or the particular modification of the poison, that there is a series of local inflammations to be set up, as in the case of scarlet fever, measles, or small-pox, inflammations which it is probable no art can prevent, and which, when moderate, render the disease both milder and safer than when the inflammations are trifling, or altogether wanting; and lastly, that the general as well as the specific actions of the poison are greatly increased by unnecessary depletion, and by whatever weakens and debilitates the body. The rules of treatment which follow from a consideration of these laws are both negative and positive. The negative are not futilely to attempt, with our present limited means, to stop the fever, and more especially by bleeding, which only the more predisposes the patient to the action of the poison; neither ignorantly to treat the patient by active measures in anticipation of the occurrence of any of the accustomed inflammations; for it is plain, from the phenomena of syphilis, that no medicine will act on the poison while yet latent, though it may control and mitigate its actions after they are set up.

The positive rules of treatment are not to anticipate any thing in fever, but to await the occurrence of each particular symptom; and inflammation being set up, to remember that it bears a specific character, and that we might as well attempt to stop the small-pox eruption as to impede its course. All, therefore, we can effect prior to the inflammation is to remove all those causes which may irritate, and consequently predispose any organ liable to be affected; or supposing the inflammation exists, to remain satisfied with so moderating its intensity, that the life of the patient may not be endangered by this particular affection; and beyond this, medicine has at present no power, except to stimulate those secretions which are in defect, and to restrain those which are in excess." 83.

As there is no known symptom, observes our author, by which we can tell when *disordered function* of the gastro-intestinal membrane ends, and change of structure begins, we may assume that, in all cases where the disease has continued even for a few hours, inflammation exists—"*An assumption which, though it may not actually be the fact, is true in forty-nine cases out of fifty.*" This is strong language, and, we apprehend, a still stronger assumption. Be this as it may, we must make room for the following mode of treating fever, as pursued at St. Thomas's Hospital.

"In a very large majority of cases diarrhœa is the only, or the principal symptom, when the alimentary canal is inflamed. The number of stools varies from three or four to eight or ten, or even to twenty, in the twenty-four hours. They are always loose, and may be indifferently green or yellow, ochre-coloured or black, and frequently contain large flakes of mucus, tinged with bile, and resembling moss-like vegetations. These stools do not furnish us with any accurate data, either as to the nature, the gravity, or the particular seat of the inflammation; for no difference has been observed in them, whether the inflammation be diffused or follicular, or whether its seat be the colon, the cæcum, the small intestines, or the stomach. It will be evident, in such a state of things, that the exhibition of a series of doses of purgative medicines can only tend to keep up an irritation, which must greatly tend to aggravate the existing inflammation; so much so, that out of ten patients treated by purgatives by Andral,* nine died. It is also certain, that even so mild a remedy as the hydrargyrus c. cretâ has little power over the diarrhœa to assuage it, and perhaps none to change the character of the stools, and to render them more healthy, until the violence of the disease be past. The whole class of astringents seldom proves efficacious; opium affecting the brain, while kino, hœmatoxylum, and catechu, increase rather than soothe the inflammation. Neutral salts, which are not purgative, as the acetate of potash, either in the state of effervescence or otherwise, are grateful to the patient, and by tranquillizing the stomach, always give relief, and are so far beneficial; but in other respects they exercise a very trifling influence over the disease.

Medicines, then, have little effect, either in controlling or subduing the inflammation of the intestinal canal in fever, or even in controlling the diarrhœa. It remained, therefore, to try what effect an almost purely local treatment would produce, and whether, by means of soothing the intestine, we might not moderate the inflammation, and in this manner produce, both directly and indirectly, more sanatory effects. Many different plans have been tried to effect this object, but that which has been found the most successful is that of enemata, consisting of barley-water and of syrup of poppies. This plan has been tried during the last seven years in a large number of patients at St. Thomas's Hospital, and with, comparatively speaking, very favourable results. The mode of treatment is as follows.

---

* Clinique Médicale, vol. iii. p. 644.

Immediately on the admission of the patient, whatever may be the stage of the disease, ten grains, or a scruple of rhubarb, are exhibited. The object of this preliminary dose is thoroughly to empty the intestines; for, notwithstanding the patient has been suffering greatly from diarrhœa, it has frequently been found, on posthumous examination, that the inflamed portion of the intestinal canal has so strongly contracted on some hard scybala, as to have retained them, and thus, contrary to all expectation, an irritating cause has existed in the intestines, which has mainly contributed to the fatal result. The bowels having been satisfactorily emptied, an enema, consisting of a pint of barley-water, together with half an ounce of syrup of poppies, has been directed to be given night and morning. This simple treatment has been continued till the patient is convalescent, and has been rarely complicated by the exhibition of any medicine whatever. Its success has been remarkable, compared with other modes of treatment, when the fever has been of any moderate degree of intensity; so much so, that in the years immediately before the cholera, out of sixty-three cases treated in this manner, only one died. The cholera was preceded and accompanied and followed by a fever of great severity, and of unusual fatality, and the treatment by enemata in those years has been by no means so successful, one being lost out of four or five. But still it has been considered, on a comparison of the deaths and recoveries under other modes of treatment, that this by enemata was on the whole the most successful. Many attempts have been made to render this simple mode of treatment more efficient by the topical application of leeches, or blisters, or mustard-poultices. There is no point, however, more delicate in the treatment of fever, or which requires so much judgment, as the interfering with its course even by apparently so slight a remedy as topical depletion. A man of about thirty years of age was brought into St. Thomas's Hospital, labouring under a severe form of fever, accompanied by slight delirium, and some bronchial inflammation. The treatment by enemata was adopted, and the case carefully watched. As soon, however, as the tongue had began to clean, and the delirium had subsided, it was thought necessary to attempt to relieve the lungs by a topical application of fifteen leeches. They were applied; and on visiting the patient the next morning, he was found labouring under such strong cerebral excitement, in consequence of this trifling loss of blood, as to be strapped down in bed. The treatment by enemata was continued, and in two days the delirium subsided, and the patient recovered. The occurrence of delirium after the application of leeches, or else of its aggravation * when it has previously existed, is so frequently witnessed, as distinctly to show, that in fever, as well as in cases of poisoning generally, in proportion as you debilitate the patient, even in the degree perhaps necessary to save a given organ, so is the nervous system generally laid only the more severely under the action of the poison. In all cases, therefore, in which there is a great predisposition to the action of the poison, and it is impossible to distinguish these cases previously to the experiment being made, leeches do harm, and retard the recovery. There are cases, however, in which delirium to a considerable, and even violent degree is kept up, reasoning from results, apparently in consequence of the sympathy existing between the brain and diseased state of

---

* A woman now lies ill of fever in St. Thomas's Hospital, from whom it was thought necessary to take 12 ounces of blood, in order to moderate the delirium; but instead of the cerebral affection being mitigated, it became more violent, so as to disturb the whole ward, and even the sister, who sleeps in an inner room; she also immediately afterwards passed her stools involuntarily. The treatment by enemata was adopted, and she so far improved that her pulse was counted below 100, and there was every prospect of her recovery; but she has been seized with erysipelas, caught from a patient lying on the opposite side of the ward, and she now lies in a very precarious state.

the intestinal canal. A nurse in St. Thomas's Hospital was seized with fever, during her attendance on a fever patient. The cerebral excitement was in this instance so great, that her screams were heard in the courts of the hospital. It became, therefore, necessary to remove her from the ward into a more private part of the building, in order that she might no longer distress the rest of the patients. It had been attempted to relieve the delirium in this case by leeches, and other topical applications to the head, but without success. Fifteen leeches were now applied to the abdomen, and the system of enemata adopted. They were successful. In a few hours the delirium subsided, and the patient ultimately recovered, though only after a long convalescence, in consequence of extensive sloughing of the back and nates. These cases are not uncommon, and I have sometimes seen delirium in fever amounting even to mania, when examination has shown only extensive ulceration of the cæcum. It is extremely difficult to determine the cases in which these different idiosyncrasies, or susceptibilities to the action of the poison, exist; but if the patient be of a full habit, and young and flushed, and the pulse one hundred and ten to one hundred and twenty in the first stage of fever, the application of leeches has in general been well borne, and given relief. On the contrary, when, as in the cholera years, the patient has been full and bloated, the countenance purple, and the sputa streaked with blood at the first onset of the disease, depletion by leeches from the abdomen, or any other part, has aggravated all the symptoms, and the patient has rapidly sunk.

It is plain, therefore, that in applying leeches to the abdomen, much is at all times put to hazard, and that the ultimate success of the case must, in a great measure, depend on the tact and judgment of the practitioner. It should also be borne in mind, that although the pain of the abdomen is in general referred to the epigastrium, the seat of the disease is, in most instances, the cæcum; and consequently, when the application of leeches is thought necessary, we embrace a much greater number of chances by applying them over the cæcum than over the epigastrium. The most undoubted symptoms warranting the application of leeches to the abdomen, is when pain is produced on making pressure over the cæcum. The state of the tongue ought not to prevent their application when this symptom is present, but they should in all cases be applied with greater reserve and caution when the tongue has changed to brown or black, showing the extreme degree in which the whole system labours under the poison.

Besides the application of leeches, blisters to the abdomen have been occasionally applied as adjuvantia to the treatment by enemata, and with good effect; but mustard poultices are to be preferred to blisters on account of their more speedily acting, of the greater surface they cover, of the heat which they impart, of the greater frequency with which they can be applied, and of their not affecting the bladder. No local application, then, has been found so useful as mustard poultices to the abdomen, and in every severe case they ought to be used once every day, or oftener, till the disease is in some degree controlled, and the patient safe.

As a general principle, therefore, a large experience has shown, that in mitigating the disordered states of the intestinal canal in typhus fever, there is no treatment so generally successful as that by syrup of poppy enemata, together with the application of mustard poultices, and occasional and moderate local bleeding. In adopting this method, we embrace two great chances: 1st, that of relieving the inflamed intestine; and 2ndly, that of relieving the brain in all circumstances in which that organ is merely sympathetically affected. The immediate operation of the enemata is to remove from the inflamed part all that irritates it, and thus to place it under circumstances the most favourable to a happy termination. Their indirect effect is to produce a general glow over the whole body, to lull the brain, and to quiet the general as well as the local irritation, and they are of the most service when they are for some time retained." 88.

When complicated with cerebral inflammation, some modification is necessary. Moderate delirium is not considered as requiring any particular treatment. When headache, however, is severe, and especially when the conjunctiva is affected, from ten to twenty leeches may be applied to the temples—and may be repeated according to circumstances. In cases where there is no headache, but where the pulse is preternaturally slow—40 or 50—leeches are necessary. As adjuvantia, cold to the shaven pate, is beneficial. Blisters, he thinks, are capricious, and little to be relied on. The lancet, he observes, is to be cautiously used when the poison acts secondarily on the mucous membrane of the air-passages. We must now conclude.

"Such is the mode of treatment a long experience has taught me to prefer to those proposed by the old masters. The exhibition of enemata as an occasional remedy is spoken of by many writers, and many physicians have attempted to avail themselves of the specific effects of bark thus introduced; but no author has made mention of any systematic use of them, founded on the conviction that the inflammation they had to deal with was long and intractable, and required a patient and persevering use of what may appear perhaps a negative mode of treatment. Success is the only criterion in medicine, and certainly this has effected the cure of a much larger proportion of cases than any other mode I have witnessed. In fevers of any moderate degree of intensity it mitigates all the symptoms, sometimes entirely prevents, but more frequently shortens the brown tongue stage. Indeed, when this treatment fails it is difficult to say what will succeed." 93.

Against facts, especially when stated without bias or prejudice, it is useless to oppose opinions. We are all aware that a great proportion of fevers will get well under no treatment at all. In the cabins and hovels of Ireland whole families are often down at the same time with typhus fever—most of them having nothing but water, or whey, or milk and water to drink; and yet a great majority survive. Whether the poppy injections of St. Thomas's Hospital act negatively or positively in the treatment of fever, we should be loth to make affidavit. Having witnessed the *apparently* good effects of purgatives—especially mild mercurials—in fever, in this and in many other climates, we confess that we should be disinclined to trust to the mode of treatment recommended in the foregoing pages. Mean time we have no doubt that amongst the fever patients of a general hospital, where misery, bad food, and various evils, moral and physical, have greatly lowered the powers of life, a treatment so inert, or rather so soothing as that employed by Dr. Williams, may possibly be that which is best adapted to such classes. A considerable number of cases are appended in illustration, drawn up by an intelligent pupil, Mr. Wegg, to which we refer our readers.

### II. On the Poison of Scarlatina.

The exanthemata are supposed to have been introduced by the Saracens about the middle of the sixth century, each depending on a distinct poison, and all causing havoc and terror through the various ranks of society to the present time. The peculiarity of these poisons consists in their generally conferring an insusceptibility to future attacks. Scarlatina and measles were long confounded together, in consequence of their having many points of resemblance, and Sydenham appears to have been the first to attempt a discrimination, but was not successful, as he only described the scarlatina sine angina—a disease of little moment. Fothergill followed, but it was Withering who first gave a luminous description of the specific differences between scarlatina and measles.

The *remote cause* of scarlatina is quite unknown. Doubtless this agent had once a local origin, but the poison is now so extensively diffused through the atmosphere of every country, that its frequent operation causes no wonder. The disease has been found to spread more fatally and freely amongst the poor than the opulent—and attacks infancy much more frequently than adult age. The individual, when once attacked, generates a poison of the same kind, which is diffused through the atmosphere, and keeps up a constant emporium of the infection. The contagion of scarlatina passes unquestioned amongst the profession, and therefore very positive proofs have not been sedulously adduced. The distance to which it extends around the person of the patient, is not ascertained. It seldom spreads in the wards of great hospitals, because the disease is chiefly confined to childhood where the susceptibility of the constitution becomes exhausted. The infecting distance, however, is generally supposed to be greater than in typhus. The *contagion* of scarlatina has been demonstrated by inoculation (Sir B. Harwood) but the disease thus produced was as formidable as that which takes place in the natural way. The propagation by fomites is universally acknowledged. The period of latency after infection is supposed to vary from a few days to ten or twelve. Many instances have been adduced where the poison produced its evident effects in two or three days. It is believed that the generation of the poison commences with the fever and before the appearance of the eruption. Dr. Willan thought that children convalescing from scarlatina, in spite of every attention to cleanliness, and change of apparel, are capable of communicating the disease (especially to other children), for two or three weeks after apparent recovery.

" *Pathology.*—The time of incubation being completed, the poison of scarlatina primarily acts on the great nervous centres, deranges their functions, and produces fever, which from its preceding the secondary or specific actions of the poison by a period of twenty-four, forty-eight, or seventy-two hours, is usually termed the primary or eruptive fever. This fever does not remit; and at the end of the period that has been mentioned, the secondary actions of the poison are set up, and the scarlet fever is fully formed. The febrile phenomena continue till after the secondary actions have terminated, when they subside, and the disease, in a great majority of cases, is at an end. In a few cases, however, the tertiary actions of the poison supervene, and the fever may or may not accompany them.

The specific actions of the poison are a peculiar inflammation of the skin, extending to the cellular tissue beneath, and producing a tumefaction of the body generally, but more especially of the hands, and this in a few cases terminates either in œdema or in mortification; secondly, of inflammation of the mucous membrane of the eyes, nose, mouth, and more especially of the throat, sometimes extending to the epiglottis, the larynx, or the trachea; and thirdly, of inflammation of the membranes, either of the brain, the joints, or of the abdominal or thoracic cavities. The inflammation of the skin is the great characteristic of the disease. It runs a given course, of from six to eight days, and with few exceptions is constantly present. The affection of the throat is also very generally present, and is only wanting in a rare form of the disease, the scarlatina sine anginâ, but its course is indefinite, and varies perhaps from eight to twenty or more days. The occurrence of the tertiary actions of the poison is only occasional, and their frequency has not been accurately determined." 122.

The disease sometimes takes on the form of a papular eruption—some-times vesicular. The appearances of the eruption of scarlatina we need not describe here, they are too well known.

Scarlatina, of every kind, is accompanied by a remarkable prostration of strength, both moral and physical—to such a degree, sometimes, as to occasion death in a few hours, or, in a few days, without any violent fever or local lesion. Scarlatina sine anginâ consists of an affection of the skin only, and is extremely mild—the primary fever being often little more than a febricula, unaggravated by the cutaneous eruption. There is also a scarlatina sine eruptione, which Dr. Sims was the first to remark. In this form the specific action of the poison is limited to one tissue, or to the mucous membrane of the mouth and fauces, the cutaneous exanthema being wanting. The angina, however, in no respects differs from the faucial affections of the scarlatina mitior and gravior. The danger is equally great as in the other varieties.

The mucous and cellular tissues of the lungs are seldom inflamed in scarlatina mitior.

*Scarlatina Gravior (Maligna of Willan.)*

"The specific actions of the poison in this form of the disease are the same as in scarlatina mitior; but the symptoms, both local and general, are more severe, the tertiary affections more frequent, and consequently the disease is more grave, and the danger more formidable.

The more remarkable symptom which distinguishes this form of the disease is the state of the tonsils. In the scarlatina mitior it has been stated that the tonsils are greatly enlarged, of a bright red, and the ulcers comparatively superficial; but in this severe form the tonsil is less swollen, but more gorged with blood, more livid in colour, while the ulcers are foul, deep, and burrowing.

The throat being less swollen than in scarlatina mitior, the absolute difficulty of swallowing is less, but the pain is greater, and the constant rejection of fluids through the nose shows how seriously the pharyngeal membrane, as also the glottis and epiglottis, are involved in the disease. It is difficult in many instances to examine the throat, for the tongue is often so tender that the slightest touch produces excoriation. The secretions from the mouth are more copious than in scarlatina mitior, and generally impregnated with the offensive sordes of the sloughs. The sloughs also are much more slowly detached than in scarlatina mitior, and when at length they are separated, an ulcer follows with a ragged edge, a foul and dirty base, and into which the finger may be thrust. These ulcers are slow to granulate, and only heal after a long, and sometimes fearful struggle; often they spread in every direction, and the parts vesicate and mortify previous to the death of the patient.

If the throat be the principal indication of the severity of the disease, the eruption affords also some peculiarities; the appearance of the cutaneous eruption is often later by some hours, and when it does appear its colour is darker and more livid, its duration more uncertain, and its distribution more capricious than in scarlatina mitior. Willan states, that he has seen it delayed till the third day, and Heberden, and Withering, have seen it delayed till the fourth day of the primary fever. The eruption also is less universal, and comes out more partially, or in scattered patches, especially about the back, the neck, the breast, and the joints. In some instances it is so fugitive that it becomes evanescent a few hours after it has appeared, returning perhaps at the end of a week, and then continuing only for two or three days. In one case Willan says it appeared for the third time, or on the seventh day from the second attack, and remained for two days.

The primary fever is usually longer than in scarlatina mitior, and sometimes precedes the eruption by three or four days. Its symptoms are more violent, and the delirium commences earlier, and is more frequently accompanied by stupor, coma, or convulsions. During the primary fever, and perhaps also during the eruptive fever, the tongue is white, but on the decline of the eruption it often changes to brown or black, or else is dry, glossy, and chapped, and then the elongated red papillæ, so remarkable and so salient when the tongue is white, disappear. The brown tongue stage is accompanied by all the phenomena of the similar stage of typhus, as low muttering delirium, subsultus tendinum, muscæ volitantes, involuntary motions, affections of the urinary bladder. From this state the patient may recover, but often when all danger seems passed, the inflamed nasal membrane discharges its fatal ichor, and causes ulceration and mortification of the mouth or cheek. In other cases, the sudden sphacelation of the whole or part of a limb terminates the patient's existence." 140.

The connexion between the gravity of the fever and the severity of the local lesions here apparent, is often wanting, especially in children. Of the tertiary actions of the poison, dropsy is amongst the most remarkable. It usually occurs about three weeks after the commencement of scarlatina. The proportion of cases in which it happens, is not ascertained. It is not the mere result of debility.

"The dropsy thus produced is usually limited to a disordered function of the cellular or serous membranes, but in a few cases it is preceded by inflammation of the pleura or peritoneum, and in either case languor, peevishness, constipated bowels, sickness and vomiting, generally precede it by several days." 141.

The œdema generally begins in the face, and often attacks no other part. When more extended, it attacks the hands oftener than the feet. The trunk, however, often becomes anasarcous, with effusion into the abdomen, or even the chest and head, though not very frequently. The urine is, at first, scanty and turbid, with frequent micturition. Afterwards it becomes copious, though still turbid. The chemical properties are altered, and heat or nitric acid shews albumen. The first appearance of œdema is accompanied by quickness of pulse, and the state of urine above-mentioned; and these are the chief phenomena; but the danger is greatly increased if effusion take place into any of the cavities, especially of the brain.

"When the ventricles of the brain are the threatened seat of effusion, the progress of the disease is so rapid that the symptoms often greatly differ from those which are seen in the more customary attacks of idiopathic hydrocephalus. The following case is an excellent exemplification of this difference. A girl eight years old, on the morning of the third day of the disease, complained of headache, which in the course of the day became exceedingly violent. In the evening she was seized with convulsions, which from the report of her mother continued for nineteen hours with scarcely any intermission; they then ceased, but returned in two hours; in this interval it was discovered that she was blind, and that the pupils were much dilated. The convulsions afterwards returned, and continued thirty-six hours, when the patient was again blind for eight hours after they had once more subsided. The œdema, which was confined to her hands and face, disappeared, while the convulsions were present. This child recovered." 143.

The next source of the greatest danger is effusion into the chest or substance of the lungs. This form is generally preceded by anasarca, and the chest is sometimes filled with remarkable celerity. A man in St. Thomas's Hospital filled so rapidly that he died before any medicine could be got to act. The œdema is often so immense, that the appearance of the patient is frightful. Infants and very young persons are almost the only sufferers from dropsy *post scarlatinam.*

We need not dwell on the *diagnosis* between scarlatina and measles. The *prognosis* is very uncertain, the mortality so much depending on the character of the epidemic, and the constitution of the individual, and even of the family. The prognosis is only grave when particular symptoms are present.

"The prognosis is unfavourable if the delirium commences, as it frequently does in children, and sometimes also in adults, a few hours after the seizure. In these cases the child often dies on the third or fourth day, and the adult on the eighth or tenth.

The tongue becoming brown, or a clean tongue with a rapid fluttering pulse, are unfavourable symptoms.

A sudden fading of the eruption, or its changing to a livid colour, are symptoms of danger.

The danger of scarlatina is also greatly increased during dentition. Pregnancy also adds to the danger, as the woman frequently miscarries. The prognosis is also extremely grave when it attacks women immediately after parturition." 145.

"The fauces becoming livid under any circumstances, or an acrid discharge from the nostrils, or else the formation of an extensive abscess of the neck, accompanied with severe purging, are all unfavourable symptoms.

The appearance of mortification in any part is commonly, but not universally fatal.

Affection of the joints is a grave, but by no means a fatal symptom.

The danger attending dropsy succeeding to scarlatina varies according to the season, to the part affected, and perhaps to the treatment. When the head or chest is affected the prognosis is always grave, and the chances are few of the patient's recovery. Dr. Cullen, when speaking of this form of dropsy, seems to consider it as of easy cure, but Plenciz and Hamilton state, that it had been more fatal in their practice than the primary disease. Mr. Wood was so fortunate as to save eight out of nine; but at the London Foundling Hospital in 1834, three were attacked, and they all died." 146.

Treatment.—In this disease the fever is intense beyond that of almost any other disease. The skin is inflamed—the fauces ulcerated—and suppuration of the joints occasionally takes place. As the inflammatory symptoms are very formidable, the question occurs, is the disease to be treated like other phlegmasiæ? Bleeding was adopted in most countries, when the disease first broke out—and with disastrous results, according to the testimonies of those times. In the hands of Morton, venesection was attended with pernicious consequences, though he recommends it. "I have seen, says he, 300 die weekly, suddenly suffocated in the second stage of the fever, and while labouring under angina or pneumonia." This is enough. Huxham abandoned venesection, and took to bark. In 1763, Dr.

Watson resumed the practice of Morton, but with very indifferent success. He lost about a sixth part of his patients. In our own times, Dr. Southwood Smith is the most strenuous advocate for general bleeding in scarlatina.

"After a decided impression has been made upon the vascular excitement by general bleeding, the application of ten or twelve leeches is of sovereign efficacy." 148.

But according to the testimony of Dr. Tweedie, out of 60 admitted into the Fever Hospital, under Dr. Smith, ten died—or about one in six—which is a very large proportion. Dr. Tweedie also adds, that instances of pleuritis, peritonitis, and synovial inflammation, were frequently observed. The amount of direct evidence against bleeding is great, but the writers rather allude to the general want of success, than to specific facts in support of their assertions. The comparative results of the venesectionary and non-venesectionary practice, however, may be gathered from the following statements.

"Dr. Sims states (p. 417), that in the year 1786 he treated upwards of 200 patients, and that out of that number he lost but two. This treatment was by gentle laxatives, mineral acids, and occasionally a little wine.

In the year 1804 scarlet fever broke out at Heriot's Hospital, in Edinburgh, when Dr. Hamilton had fifty of the boys placed under his care, and of these three only died, and those of the secondary symptoms, or dropsy, and so rapid was the watery effusion that it filled the cellular membrane, and inundated every cavity within thirty-six hours from the attack, and the boys died labouring under symptoms denoting ascites, hydro-thorax, and hydrocephalus. In the treatment 'I employed purgative medicines fully. The effect was favourable; the fæces were hard, generally of a black or greenish colour, and fœtid, and sometimes of the colour and consistence of clay, and less fœtid. In proportion to the evacuation of these fæces relief was perceptible.'

In the year 1820 scarlatina broke out at Alford, in Aberdeenshire, and out of 160 cases treated by Dr. Murray, only sixteen died, three of them women, immediately after delivery. This appears to have been a severe form of the disease, and was in the latter stages attended with 'a swelling of the wrists and hands, and less commonly of the knee joints, and other articulations, attended with much pain, and a feeling of the want of the power of motion. In the treatment bleeding was but rarely practised.' (P. 347.) 'Purgatives were not omitted, but I was somewhat disappointed in the degree of benefit derived from them. Emetics were frequently employed, but they only gave relief to the throat, obstructed by swelling or mucus, and their effect in this way was generally very great.' 'Latterly, however, they have been exhibited only in very urgent obstructions of the throat, for in a few cases they left distressing and long continued sickness, and I think I have known them increase the tendency to sensorial derangement.' The affections of the head were met by shaving the scalp, blistering the nape of the neck, putting sinapisms to the feet, and warm fomentations to the legs. The affections of the joints were commonly very distressing, but were in general removed by warm fomentations; stimulants were not used till late, and were generally unsuccessful, though he admits there are cases in which they are admissible.

In the year 1832-3 scarlet fever again broke out at Heriot's Hospital, when forty-five of the boys were seized, and placed under the care of Dr. Hamilton and Mr. Wood, and only one of the forty-five died, although nine fell ill of dropsy. The treatment (p. 37) of the disease, in its primary form, was extremely simple. In some of the earlier cases emetics of ipecacuanha were administered at the commencement of the complaint, but in the latter cases purgative medicines were used in preference, and repeated almost daily, till the fever had subsided, when they were continued at intervals, more or less short, till the patient was considered perfectly cured. The purgatives employed were compound powder of jalap, jalap and calomel, supertartrate of potash, inf. sennæ, Epsom salts, and compound colocynth pills, varied according to circumstances.

In 1834 scarlet fever prevailed also at the London Foundling Hospital, and upwards of 100 of the children were seized with it. Out of this number only three died, and those of the secondary affection, or of dropsy. The treatment in this case was principally by mineral acids, small quantities of wine, jellies, and a nourishing, but antiphlogistic diet.

Such are the results that have been obtained by the practice of bleeding, as well as by abstaining from it, in the cure of scarlet fever; and if we compare them, the results will stand thus:—

| | | | | |
|---|---|---|---|---|
| Of | 121 | treated at the Foundling Hospital in 1786 by bleeding | 19 | died |
| — | 60 | ............ London Fever Hospital in 1829 ...... | 10 | — |
| | 181 | | 29 | |

Or nearly 1 in 6.

| | | | | |
|---|---|---|---|---|
| While of | 200 | treated by mineral acids and wine, &c. ........... | 2 | died |
| — | 160 | ........ purgatives and emetics ............... | 16 | — |
| — | 50 | ........ ditto ............... | 3 | — |
| — | 45 | ........ ditto ............... | 1 | — |
| — | 100 | ....... mineral acids and wine ............... | 3 | — |
| | 555 | | 25 | |

Or nearly 1 in 22.

It seems therefore proved, that one in six has died after bleeding, while only one in twenty-two has died after a milder, if not a directly opposite mode of treatment; and the conclusion which inevitably follows is, that the chances of recovery are diminished by the practice of bleeding in the ratio of nearly four to one, as compared with the chances supposing the patient not to have been bled." 152.

This bad success led to an extensive use of bark in scarlatina, its advocates enumerating the names of De Haen, Sauvages, Kennedy, Johnston, Huxham, Cullen, Clarke, Perceval, &c. The late Dr. Powel uniformly treated scarlatina with bark in Bartholomew's Hospital, whatever was the stage or the symptom of the disease!! It is stated by our author that all the cases thus treated recovered! Credat Judæus. Dr. Witheringham gave bark a fair trial at Birmingham, but with very different success from that observed by Dr. Powel. The following is the attempt of Dr. Williams to reconcile conflicting testimonies, and to establish, if possible, "some general rule by which this always greatly complicated, and too often fatal disorder may be best combated."

"The scarlatina sine angina is a mild form of the disease. It is sufficient to confine the patient to the house—to strictly enjoin a milk diet—to regulate his bowels, and above all things to avoid, according to the precept of Sydenham, the nimia diligentia medicorum, for no other medicine is necessary than gentle laxatives to regulate the bowels. The disease thus treated is uniformly mild, and when the rash declines, the fever subsides, and the disease is at an end.

The treatment of the scarlatina sine eruptione is the same as that of the two following varieties, or that of the scarlatina mitior and of the scarlatina gravior.

The scarlatina mitior is a form of the disease, it has been stated, which differs from scarlatina gravior principally in the circumstance of the greater swelling of the tonsils and uvula, of their being more superficially ulcerated, and indeed from their presenting altogether an inflammation of a more sthenic character.

It was in this form of the disease, 'when the tumefaction of the fauces was such that the patients could not swallow but with the utmost difficulty,' that Dr. Withering found sudorifics, cordials, and alexipharmics, 'have but little to do in the cure of scarlatina anginosa,' and that bark poured down with an unsparing hand produced such consequences 'as would not justify a continuance of its use.' It was in this form of the disease, when 'the most frequent appearance was a great swelling of the tonsils,' that Dr. Sims, who treated so successfully the other forms of the disease by gentle laxatives a draught containing twenty drops of sulphuric acid, together with a little wine—found if this cordial system was persisted in, or increased with a view to keep up a sinking pulse, many vexatious or even dangerous consequences ensued. For 'a new fever, often more violent than the first was raised—a great swelling and inflammation of the tonsils or parotids, with acute pain, came on, and the scarlet eruption appeared as copiously as before. I always,' he adds, 'subtracted somewhat from the wine and cordials, and quickly prohibited them entirely, diminishing at the same time the spirit of vitriol, which seemed now unnecessary, and giving rhubarb in smaller doses, and relying, during the period of amendment, upon gentle nourishing diet and broths as the only medicines, except when some particular symptom seemed to require attention.' (P. 423.) The accidents observed by Sims and Withering, and which led them to doubt of the efficacy of bark in any form of scarlet fever, only occur according to my observation when scarlatina mitior is treated by bark, for although the ultimate result is generally successful, still for a time the fever is often greatly aggravated, the troubles of the throat increased, the convalescence tedious, and consequently this form of the disease appears to admit of a middle course of treatment, or of one less debilitating than by general bleeding, and less powerfully stimulant than by bark or quinine.

The treatment then of scarlatina mitior is first to tranquillize the stomach and to allay its inverted action, when vomiting exists, either by small doses of the sulphate of magnesia, or by the effervescing draught, medicines which according to the state of the bowels may be exhibited every four or six hours. As soon as this object is effected, and it is ascertained that the tonsils have acquired the peculiar character which marks this form of the disease, and are enlarged and swollen, the practice is to relieve them by local bleeding, and twelve to fifteen leeches should be applied to the throat and allowed to draw freely. The trifling loss of blood thus sustained does not impair the general strength of the patient, while it greatly reduces the swelling of the tonsils, and prevents their becoming permanently enlarged. Another advantage is also gained by the application of leeches to the throat, namely, that they relieve the head and tranquillize the delirium, for it is a law of diseases depending upon morbid poisons, that by relieving the part specifically acted upon we relieve the brain. A very marked instance of this twofold relief was lately observed in a young woman of twenty years of age, who laboured under scarlatina mitior, accompanied with severe headache and very active delirium. These symptoms were attempted to be relieved by the repeated application of leeches to the temples, but without success. A similar application of leeches was now made to the throat, when not only was the affection of the tonsils relieved, but the delirium immediately subsided. The relief obtained in this case was remarkable, and as a general principle it follows in similar instances. When the disease is severe it is occasionally, but not often, necessary to repeat the leeches.

The tonsils having been relieved, the fever may now be permitted to run its course little influenced by medicine, and the patient only refreshed by the occasional exhibition of the saline draught, so grateful to his parched mouth and feverish state. For if in these cases we stimulate the patient we only bring back the tumefaction of the tonsils; while, on the contrary, if we take more blood, we hazard the producing the more serious accidents incident to scarlatina gravior. The medicines, therefore, that have been mentioned should be persevered in till the disappearance of the eruption, the healthy granulations of the throat, and the decline of the fever, give a certain evidence of a state of convalescence. At this point, perhaps, some tonic medicine is desirable,* and prepares the patient once more for the fullest enjoyment of health. This is the most successful treatment of the scarlatina mitior.

The scarlatina gravior is characterised by the less swollen state of the tonsil —by its being more gorged with blood—by its greater lividity of colour, and by the ulcers being deeper and more spreading, and by the slough being fouler than in the former variety. In this disease also a greater tendency exists in different parts to run into mortification. These different circumstances seem to point to the necessity of adopting a more stimulating plan of treatment, and one more calculated to support the powers of the constitution. The danger, indeed, of permitting this disease to run its course without the aid of medicine may be instanced by the following cases:—A child of about four years of age was seized with scarlet fever. The disease was not interfered with, but permitted to run its course without the aid of medicine. On the decline of the eruption, a discharge took place from the nostrils, the lip inflamed became gangrenous, and the child died in consequence. A man was received into St. Thomas's Hospital with the eruption out upon him, and attention was alone paid to the state of his bowels. In a few hours the right lower extremity mortified from the heel to the hip, and it is unnecessary to state that he also died.

A tonic treatment, therefore, appears necessary in this form of scarlatina, and since it has been proved that bark or quinine is not a specific remedy, it is open to us to adopt either a treatment by that medicine, or else by any other mineral or vegetable tonic we may think preferable. When the sulphate of quinine is preferred, the usual dose for an adult is five grains every four or every six hours, and as the powers of easy deglutition are greatly impaired, it is best exhibited out of the infusion of roses, in which it is dissolved by aid of the acid contained in that preparation. In children, of course, the dose must be diminished in proportion to their age. This medicine, when it sits lightly on the stomach, is all that is essentially necessary in the cure of this form of the disease, nor should the presence of delirium prevent its exhibition.

Still it frequently happens that quinine is rejected by vomiting, or that in many instances it is impossible to induce children to take so bitter a medicine without using a most distressing degree of violence, and consequently we are often obliged to look for an equivalent; and it has been proved, by frequent experiment, that wine may be substituted for quinine, both in the child and the adult, without diminishing the efficacy of the treatment, or endangering in any degree the result. Indeed wine being much more easily digested than quinine, the substitute is not only more agreeable, but to a certain extent more advantageous. The quantity for an adult is from four to six ounces, and in the child about half that quantity in twenty-four hours. There may indeed be cases in which it may be necessary to increase those quantities, but they are rare. The wine may be either port or sherry, and is best taken with sago, or drank diluted with water, and should be exhibited in small portions at short intervals in the course both of the day and night. The existence of delirium does not contraindicate the treatment by wine, for most commonly that symptom is merely a

* "℞. Infusi Aurantii ℥ iss., c. Ræ Aurantii ʒ iss., or from five to ten grains of salicine ter die; or perhaps, what is still more agreeable, the patient may be indulged with three or four ounces of wine daily."

derangement of function, and may be disregarded. The wine should be continued till the patient is decidedly convalescent, and even perhaps for some time afterwards. But while pursuing this treatment it is necessary that the patient's bowels should be strictly attended to, and be gently and daily relieved." 158.

When inflammation takes place into the joints, stimulants are to be avoided, though bleeding will seldom be necessary. A brisk action is to be kept upon the bowels, and hyoscyamus may be given to allay pain.

The effusions into the cavities are serious matters, and our author strongly recommends bleeding as soon as œdema appears in the face, especially if accompanied by headach. The rest of the treatment consists in brisk purgation, the choice of purgatives being left to the practitioner. Supertartrate of potash, in ʒj. doses, is a favourite of our author's. In our own practice we have seen better effects from small doses of elaterium. When the fluid is evacuated, Dr. W. recommends salicine, "which is an excellent tonic and diuretic, and may be given in five-grain doses three times a day, or else ten grains of the tartrate of iron." Emetics and purgatives are not commended by Dr. W., and neither is cold affusion, as employed by Dr. Currey. Ablution or sponging of the face and hands by cold or tepid vinegar and water is a practice, grateful to the patient, though, he thinks, of little importance as influencing the final result. Blisters are condemned, and gargles are inapplicable to the cases of children. Slightly acidulated ones are all that Dr. W. uses till the active stage has passed, when stimulants may be employed. The best is probably the acidulated infusion of roses sweetened with honey or sugar.

As to prevention, our author has no faith in *disinfectants*—except ablution and ventilation. As for Hahnemann's Belladonna theory, it is justly scouted by our author, as one of the tom-fooleries of homœopathy. If any effects ever resulted from this or any other preventive or disinfectant, they have, in all probability, been the products of moral courage or sense of security—powerful means of resisting contagion of every kind.

We have thus given a very full analysis of this section of Dr. Williams's work, because the subject is important—the matter practical—and the author experienced. If these reasons are not satisfactory, we know of none other more valid.

